# Integrating artificial intelligence with nanodiagnostics for early detection and precision management of neurodegenerative diseases

**DOI:** 10.1186/s12951-025-03719-x

**Published:** 2025-10-13

**Authors:** Youssef M. Hassan, Ahmed Wanas, Ayat A. Ali, Wael M. El-Sayed

**Affiliations:** 1 Department of Zoology, Faculty of Science, Ain Shams University, Abbassia, Cairo, 11566 Egypt; 2 Department of Biochemistry, Faculty of Science, Ain Shams University, Abbassia, Cairo, 11566 Egypt; 3 Biotechnology program, Faculty of Science, Ain Shams University, Abbassia, Cairo, 11566 Egypt

**Keywords:** Biomarker detection, Blood–brain barrier, Early diagnosis, Nanomedicine, Targeted drug delivery, Theranostics

## Abstract

**Background:**

Neurodegenerative diseases—including Alzheimer’s, Parkinson’s, and amyotrophic lateral sclerosis (ALS)—as well as autoimmune disorders with neurodegenerative features such as multiple sclerosis (MS), present an escalating global challenge. Current diagnostics often detect pathology too late, and most treatments focus on symptom relief rather than disease modification. There is an urgent need for tools that enable early detection and precision-targeted intervention.

**Main body:**

Nanotechnology offers unique advantages in this space, enabling early molecular detection, targeted drug delivery, and theranostic systems. Engineered nanocarriers, biosensors, and responsive nanodevices are being tailored to disease-specific features such as oxidative stress in AD or neuroinflammation in MS. Yet, issues like biocompatibility, clinical scalability, and long-term safety remain barriers to translation. Artificial intelligence (AI) enhances nanomedicine by improving biomarker sensitivity, stratifying patients, and enabling predictive disease modeling. From AI-guided nanoparticle design to closed-loop delivery systems and digital twin models, these technologies work synergistically to support real-time, personalized care. Still, critical challenges—including algorithmic bias, lack of explainability, heterogeneous datasets, and limited regulatory clarity—impede clinical integration. Additionally, high system complexity and cost risk excluding low-resource settings unless inclusive, scalable alternatives are pursued.

**Conclusion:**

The convergence of AI and nanotechnology is reshaping neurodegenerative disease care, moving from reactive to proactive, personalized neurology. Realizing this promise requires cross-sector collaboration, ethical foresight, and translational rigor to ensure these innovations are safe, equitable, and accessible to all patients.

**Graphical Abstract:**

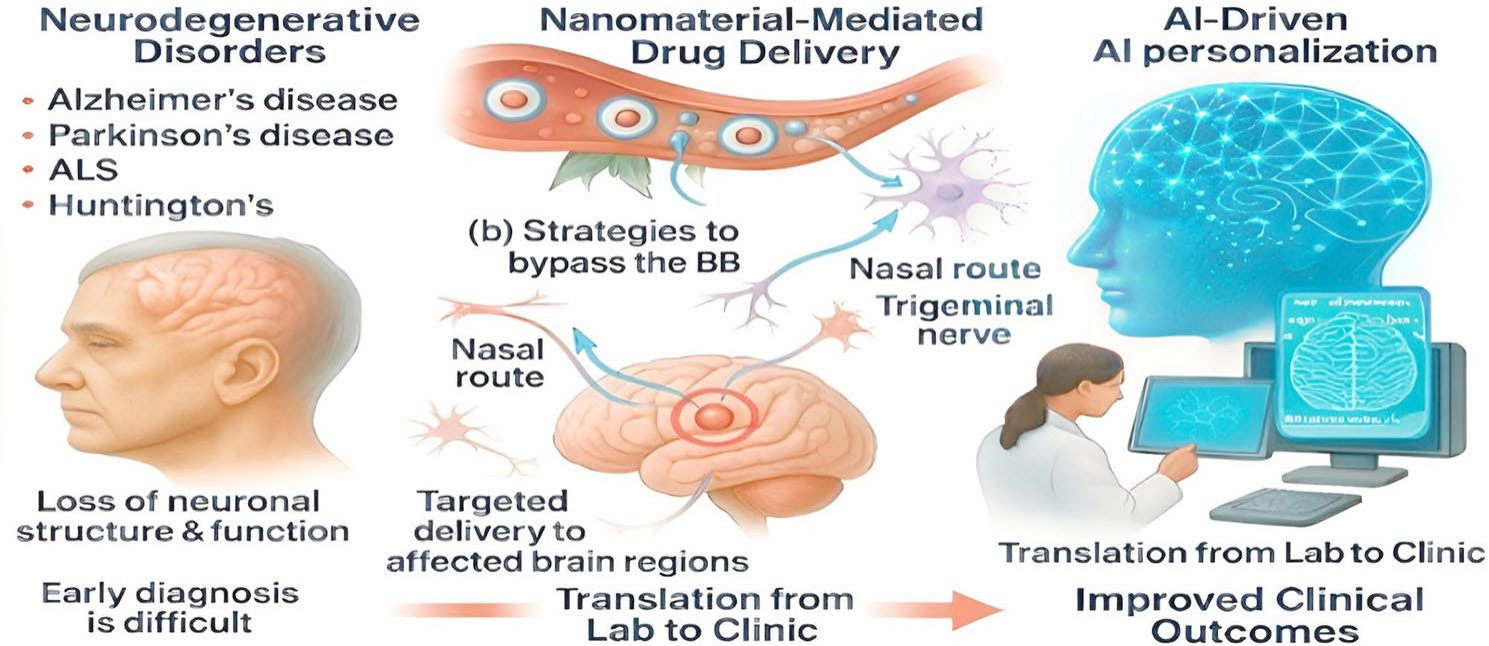

## Introduction

Alzheimer’s disease (AD), Parkinson’s disease (PD), and amyotrophic lateral sclerosis (ALS) are major neurodegenerative diseases (NDDs), while multiple sclerosis (MS) is an autoimmune/inflammatory disorder with prominent neurodegenerative features. Together, these conditions present an escalating global health challenge. Despite differing etiologies, these disorders share common pathophysiological mechanisms—including protein misfolding, oxidative stress, mitochondrial dysfunction, and neuroinflammation—that progressively erode neuronal integrity and function. Current treatments remain largely symptomatic, with no curative or disease-modifying options approved for most patients [1]. As populations age, the demand for earlier diagnosis and more precise therapeutic intervention continues to grow.

However, the ability to intervene early is limited by technological and biological barriers. Traditional diagnostic modalities—such as neuroimaging, cerebrospinal fluid (CSF) analysis, and neurocognitive assessments—often detect disease only after significant neuronal loss. Therapeutic delivery is further complicated by the BBB, which restricts the entry of most biologics and small molecules into the CNS [2].

Nanomedicine has emerged as a potential enabler of earlier diagnosis and targeted intervention. Nanoparticles (NPs) offer unprecedented access to CNS targets by leveraging BBB-penetrating strategies and tunable surface chemistries. They can simultaneously carry drugs, imaging agents, and targeting ligands, creating multifunctional theranostic platforms that promise to enhance treatment efficacy while minimizing systemic toxicity [3, 4]. Additionally, nanostructured biosensors are enabling ultra-sensitive detection of early-stage biomarkers in blood and saliva, using technologies like surface-enhanced Raman scattering (SERS) and localized surface plasmon resonance (LSPR) to outperform traditional ELISA [5].

Yet, most of these advances remain at the preclinical stage. A critical analysis reveals persistent challenges: reproducibility issues, poor scalability of complex nanocarriers, and unclear clinical validation pathways. For example, while dozens of nanoparticle formulations show strong BBB penetration in rodent models, very few demonstrate meaningful translation in humans due to interspecies differences and systemic clearance [6].

AI introduces a second axis of innovation, particularly in interpreting high-dimensional data from neuroimaging, proteomics, and wearable sensors. Deep learning models have shown promise in identifying preclinical disease patterns and stratifying patients into molecular subtypes for personalized therapy [7]. In parallel, AI accelerates nanoparticle design via materials informatics, predicts BBB permeability, and enables adaptive feedback systems that monitor disease markers in real-time to guide therapy [8].

However, enthusiasm around AI-driven solutions often overshadows unresolved limitations: lack of model interpretability, algorithmic bias, and a scarcity of high-quality, diverse training datasets. Furthermore, the ethical and regulatory frameworks for AI-integrated diagnostics and therapeutics are still underdeveloped, raising concerns about clinical adoption and equitable access [9, 10].

Instead, it critically evaluates how the convergence of AI and nanotechnology may enable a shift from reactive to predictive neuromedicine—and what bottlenecks must be addressed to achieve that vision. We begin by outlining shared molecular features and diagnostic gaps across major NDDs. We then explore nanotechnological advances for CNS drug delivery and biosensing, followed by a synthesis of AI applications in disease prediction and therapeutic optimization. Finally, we discuss real-world barriers to translation—including GMP compliance, regulatory ambiguity, and data privacy—and propose integrative pathways for clinical implementation.

By framing this convergence through the lens of translational readiness, we aim to offer not just a survey, but a roadmap for advancing personalized, proactive, and precision-based approaches to neurodegenerative disease management.

### Methodology

This narrative review synthesizes recent advances in nanomedicine for the diagnosis and treatment of NDDs. A targeted literature search was conducted using PubMed, Scopus, and Web of Science to identify peer-reviewed studies published between January 2021 and May 2024. Search terms included combinations of brain-targeted NPs, targeted drug delivery, neurodegeneration, blood–brain barrier, neuroimaging, nanosensors, nanotherapeutics, and biomarker detection, with MeSH terms and Boolean operators applied to enhance sensitivity. The focus was on ALS, PD, AD, Huntington’s disease, and related CNS disorders.

Studies were included if they investigated nanotechnology for monitoring, diagnosing, or treating NDDs and reported original in vitro, in vivo, or clinical data on nanomaterials such as inorganic nanocarriers, liposomes, polymeric NPs, dendrimers, or exosomes. Articles were excluded if they lacked clinical or neurobiological relevance, contained no experimental or translational data, or were preprints, theses, book chapters, editorials, or conference abstracts. Only English-language studies were reviewed to ensure consistency in technical interpretation.

To reduce bias, studies were assessed for methodological transparency, statistical rigor, and relevance, with preference given to those using multiple biological models, clear controls, and validated biomarkers. Potential conflicts of interest and funding sources were noted to maintain critical evaluation. While the exclusion of non-English publications may introduce some bias, it facilitated consistent analysis of the technical and translational implications of nanomedicine in neurodegenerative disease management.

## Neurodegenerative diseases (NDDs) overview

NDDs involve the progressive loss of neurons and glial cells in specific regions of the CNS. Although AD, PD, ALS, and MS differ in molecular triggers and clinical presentation, they converge on shared pathological features: misfolded protein accumulation, oxidative stress, neuroinflammation, mitochondrial dysfunction, and eventual neurodegeneration. These overlapping mechanisms offer opportunities for platform-based therapeutic strategies, yet also complicate diagnosis and intervention due to their biological complexity and patient heterogeneity.

Nanomedicine is well-positioned to address these challenges through targeted drug delivery and biomarker detection, especially when paired with AI-driven models for patient stratification and treatment optimization. However, as the following disease-specific sections reveal, while numerous nanoparticle systems show promise in preclinical models, clinical translation remains rare—highlighting a gap between scientific innovation and therapeutic reality.

### Alzheimer’s disease (AD)

Alzheimer’s disease (AD) is characterized by extracellular amyloid-β (Aβ) plaque deposition and intracellular neurofibrillary tangles of hyperphosphorylated tau. These aggregates trigger synaptic dysfunction, oxidative stress, and neuroinflammation, ultimately leading to irreversible neuronal loss. Fluid biomarkers such as CSF Aβ1–42, p-tau181, and neurofilament light chain (NfL), along with emerging blood-based assays, have advanced early detection capabilities; however, their integration into clinical workflows remains limited due to assay variability, accessibility issues, and regulatory hurdles [11, 12].

**Fig. 1:**
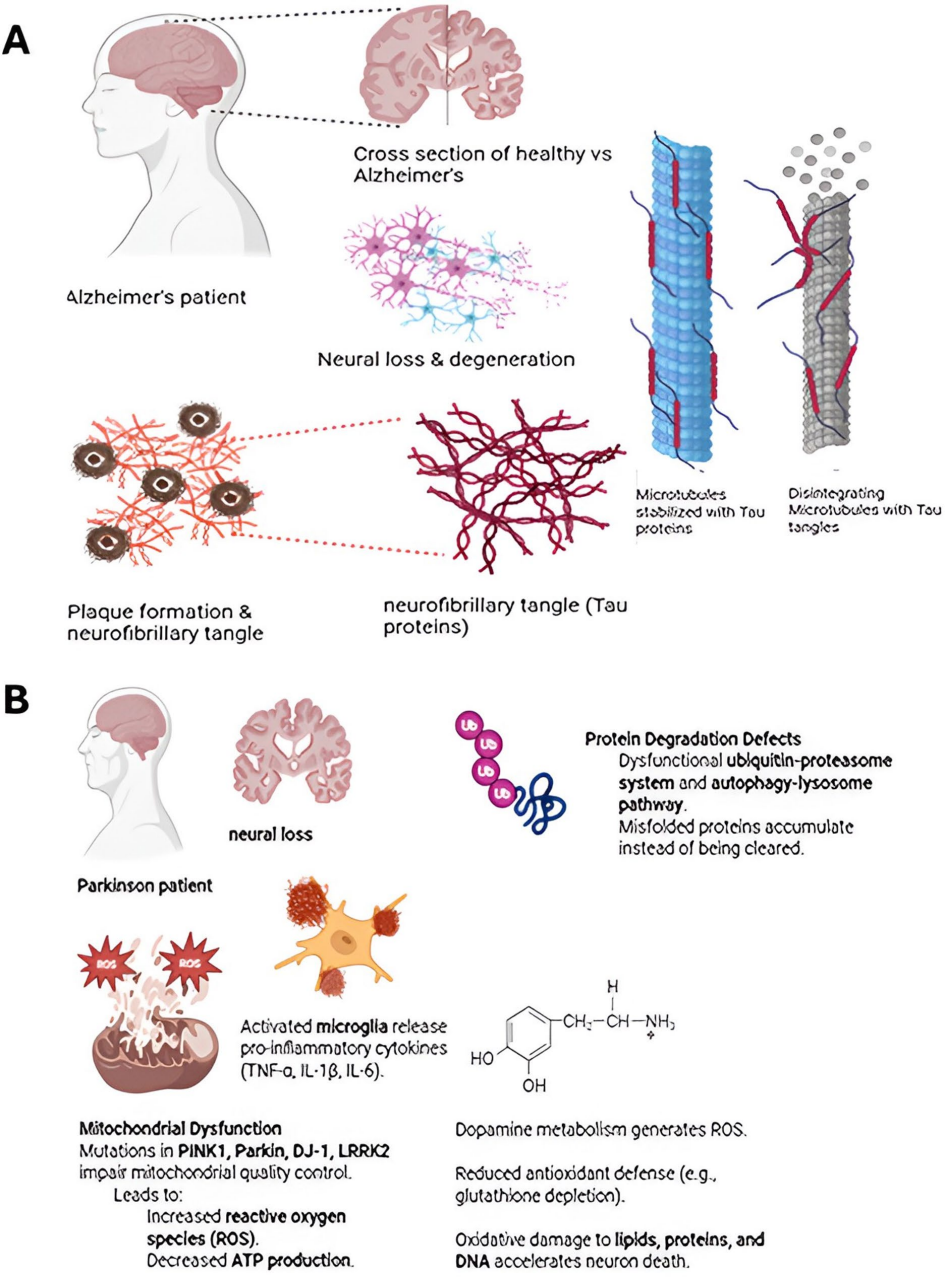
Schematic representation of pathophysiological mechanisms in major neurodegenerative diseases. (**A**) Alzheimer’s disease (AD): Abnormal cleavage of amyloid precursor protein (APP) produces amyloid-β (Aβ) peptides that aggregate into extracellular plaques. Concurrently, hyperphosphorylated tau accumulates intracellularly to form neurofibrillary tangles. These processes contribute to synaptic dysfunction, hippocampal atrophy, and progressive neurodegeneration. (**B**) Parkinson’s disease (PD): Misfolding and aggregation of α-synuclein into Lewy bodies occurs within dopaminergic neurons of the substantia nigra. This disrupts mitochondrial function and proteostasis, ultimately causing dopaminergic neuronal loss and motor impairmen

Aβ fibrillation is considered a central pathogenic event in AD, making its removal and dissolution a major therapeutic goal. While numerous Aβ inhibitors have been developed, most suffer from low binding affinity, resulting in suboptimal outcomes. One promising approach is the Aβ-sequence-matching strategy, which enables the design of dissociable nanosystems—such as B6-PNI NPs—with enhanced Aβ-binding affinity. These nanosystems can both promote the dissolution of amyloid fibrils and modulate the in vivo fate of Aβ to facilitate its clearance [13], (Fig. 1). Beyond inhibition or fibril dissolution, emerging nanoparticle-enabled targeted protein degradation platforms offer a novel route to eliminate extracellular pathological proteins, leveraging proteolytic mechanisms to directly dismantle amyloid and tau aggregates [14].

Beyond amyloid-targeting strategies, several NP platforms aim to improve CNS delivery of anti-inflammatory or anti-aggregative agents. For example, ROS-sensitive, RAGE-targeted NPs have shown improved brain accumulation and cognitive performance in AD mouse models [15]. However, such promising results are often based on idealized animal models that fail to fully capture the complexity of human disease, particularly regarding amyloid deposition patterns, immune microenvironments, and BBB dynamics.

Historically, most AD nanotherapies have targeted amyloid clearance or tau inhibition—approaches that have yielded mixed or negative results in late-stage clinical trials. This raises the question of whether these molecular targets remain viable in heterogeneous patient populations. Future research should critically evaluate the translational potential of NP-based therapeutics, considering disease variability, co-pathologies, and real-world treatment timelines.

While recent β-amyloid–targeting antibodies have received regulatory approval, their therapeutic benefit remains constrained by pathological variability among patients. Gene therapy has emerged as another avenue for personalized intervention, offering the potential to address underlying genetic and molecular drivers of AD. Yet, in the CNS, gene therapy faces substantial obstacles—including patient-to-patient heterogeneity, incomplete understanding of druggable targets, and delivery challenges. With their precision delivery capabilities, nanomedicine platforms could help overcome some of these barriers, providing a flexible foundation for targeted and personalized gene therapy in AD [16].

### Parkinson’s disease (PD)

PD is marked by α-synuclein aggregation and dopaminergic neuron loss in the substantia nigra, leading to motor and cognitive impairment. Biomarkers remain limited and variable in consistency, with α-synuclein isoforms and NfL under investigation but not yet clinically validated [17].

Nanocarriers for PD have explored dopamine replacement, gene therapy, and neuroprotective payloads. Notably, albumin/PLGA-based NPs designed for dopamine delivery have shown significant functional improvement in animal models by leveraging RMT and sustained release [18]. While this demonstrates rational design principles, translating these benefits to human systems remains a challenge due to potential immunogenicity, variable receptor expression, and rapid systemic clearance (Fig. 1).

Gene-targeted nanotherapies (e.g., siRNA, miRNA, CRISPR/Cas9) offer exciting potential, particularly for monogenic PD variants, but pose risks related to off-target effects, delivery efficiency, and ethical concerns around long-term gene modulation. Comparatively, fewer AI-guided or adaptive nanocarrier systems have been developed for PD relative to AD, highlighting an area for future integration.

### Multiple sclerosis (MS): a neuroinflammatory disease with degenerative features

MS is primarily considered a chronic autoimmune and inflammatory disorder of the CNS, characterized by demyelination, neuroinflammation, and episodic relapses. However, in its progressive forms, MS exhibits sustained axonal degeneration, cortical atrophy, and irreversible neuroaxonal loss—hallmarks shared with classical NDDs. These overlapping features increasingly justify MS’s inclusion in discussions of neurodegenerative mechanisms and interventions.

Clinically, biomarkers such as NfL, glial fibrillary acidic protein, and oligoclonal bands aid in disease monitoring and prognosis. Therapeutic approaches are shifting toward agents that modulate immune response, promote remyelination, or preserve neuronal integrity [19].

Nanomedicine in MS is often deployed in conjunction with regenerative strategies, particularly stem cell therapies. NPs are used to enhance targeted delivery, support cell survival, and guide differentiation. Techniques such as magnetically guided cell homing, nano-scaffold-mediated regeneration, and exosome-nanoparticle hybrids for immunomodulation show promise [20, 21]. However, these approaches remain technically demanding. Challenges include ensuring the stability of cell–nanoparticle interactions, avoiding immune rejection, and scaling production for clinical application.

Unlike in AD or PD—where nanotechnology often aims to directly modify pathology—MS nanomedicine tends to play an adjunctive or enabling role, especially in regenerative or immunomodulatory therapies. This distinction adds complexity to clinical translation, as therapeutic outcomes depend on multifactorial interactions between nanomaterials, immune responses, and host tissues.

### Cross-disease synthesis: opportunities and bottlenecks

Despite the disease-specific differences described above, several cross-cutting themes and limitations emerge that highlight both the potential and the complexity of nanomedicine in neurodegeneration. Across AD, PD, and MS, oxidative stress and chronic inflammation play central roles in neuronal injury and are commonly targeted using ROS-sensitive, anti-inflammatory nanoparticle systems. This convergence suggests that a subset of nanotherapeutic designs may be broadly applicable across multiple neurodegenerative contexts, particularly in early disease stages where shared pathological pathways dominate.

Similarly, many of the most advanced nanocarrier systems aim to address the challenge of BBB penetration, employing strategies such as ligand-mediated transcytosis or biomimetic camouflage. These tactics have been replicated in different diseases with varying degrees of success, but their translation to human subjects remains hindered by species-specific differences in BBB structure, receptor expression, and immune response.

Theranostic systems—nanoplatforms combining drug delivery and diagnostic functionality—are another area of conceptual overlap. While these systems hold great promise for real-time monitoring and adaptive therapy, they also represent some of the most complex and least standardized technologies in the field, often requiring integration of diverse materials, imaging agents, and release triggers. Their potential is highest in precision medicine settings, yet scalability, regulatory acceptance, and cost-effectiveness remain unresolved.

At a broader level, most nanotechnologies described here still operate in isolation from biomarker data or AI interpretation. Truly adaptive and personalized treatment systems—where AI-guided feedback modifies nanoparticle behavior in real-time—are still aspirational. The technologies exist in parallel but are rarely integrated, revealing a missed opportunity that this review seeks to spotlight.

In summary, while nanomedicine offers exciting solutions tailored to the unique pathology of each NDD, its widespread clinical use is constrained by recurring bottlenecks: species translation gaps, challenges in manufacturing reproducibility, lack of standardized biomarker integration, and the nascent state of AI-nanomedicine convergence. Understanding these limitations—and designing technologies to overcome them—will be crucial for translating laboratory breakthroughs into accessible clinical solutions.

## Limitations and opportunities in current neurodegenerative disease management

Despite notable advances in our understanding of NDD pathogenesis and biomarker discovery, the clinical management of these disorders remains insufficient. This shortfall arises from a complex interplay of diagnostic limitations, treatment delivery challenges, and a lack of personalization in care strategies. However, when analyzed across diseases, these shared limitations also reveal common intervention points where nanotechnology and AI could offer transformative solutions—if their development accounts for real-world translational constraints.

### Delayed and difficult diagnosis

One of the most pervasive challenges in NDD management is delayed diagnosis. In AD, PD, and other NDDs, pathological processes such as Aβ aggregation, tau hyperphosphorylation, or α-synuclein deposition often begin decades before clinical symptoms emerge. By the time motor or cognitive symptoms become evident, extensive and irreversible neuronal loss has occurred.

Current diagnostic tools—predominantly neuroimaging and clinical assessments—are poorly suited for early-stage detection. Their limited sensitivity at preclinical stages diminishes the potential for disease-modifying interventions. While blood-based biomarkers offer promise, particularly for AD (e.g., plasma p-tau181, Aβ42/40 ratios), these assays remain under validation and are not yet widely implemented [22].

In diseases like MS, relapses can be detected through imaging, but subclinical disease progression often goes unnoticed until functional impairment occurs. These diagnostic gaps underscore the need for biosensing platforms that are not only highly sensitive but also adaptable to real-time monitoring and patient-specific baselines.

### Invasiveness and scalability barriers

Many gold-standard diagnostics—including CSF analysis via lumbar puncture and PET imaging for Aβ or tau—are invasive, costly, and inaccessible for population-wide screening or routine longitudinal monitoring. Their use is limited to select research centers and high-resource clinical settings. This severely restricts early detection efforts and excludes vulnerable populations from timely intervention.

Nanobiosensors, capable of detecting ultra-low concentrations of disease biomarkers in blood, saliva, or tear fluid, have the potential to decentralize and democratize diagnostic access. However, few such systems have been validated in large cohorts, and many still require signal amplification platforms or microfluidic integration that increase complexity.

### Biomarker ambiguity and lack of specificity

While NfL and other emerging biomarkers provide useful indicators of neuroaxonal damage, they often lack disease specificity. Elevated NfL levels are seen in AD, PD, ALS, and MS alike, complicating efforts to distinguish between conditions or track disease-specific trajectories. AI-based biomarker profiling could help resolve these ambiguities by combining multi-omic and imaging data to build probabilistic disease models. Still, this promise remains largely theoretical due to the paucity of harmonized datasets.

### Therapeutic delivery challenges

Even when effective therapies are identified in vitro, their delivery to target CNS regions remains a major bottleneck. The BBB significantly limits drug permeability, and many systemically administered agents fail to reach therapeutic concentrations in the brain. Nanocarriers offer potential solutions via receptor-mediated transport and controlled release mechanisms, but their clinical success is limited by variability in BBB receptor expression, nanoparticle clearance, and off-target immune responses.

While promising examples exist—such as dopamine-loaded NPs for PD or RAGE-targeted systems in AD—most remain in preclinical or early-phase trials, with efficacy and safety in humans yet to be established. Furthermore, regulatory approval for multifunctional nanocarriers remains complex, especially for platforms integrating diagnostics and therapeutics.

### Lack of personalization and dynamic monitoring

Despite significant heterogeneity in genetic profiles, disease mechanisms, and treatment responses, most NDD management remains standardized and reactive. Patients are rarely stratified based on molecular subtypes, and treatments are seldom adjusted in real time based on evolving biomarker profiles.

This disconnect presents a key opportunity for AI-integrated nanosystems. By enabling real-time biomarker sensing and data-driven interpretation, such systems could deliver adaptive therapies that evolve with disease progression. However, clinical examples of this closed-loop paradigm remain scarce, and current regulatory frameworks are not yet equipped to evaluate dynamically updating treatment platforms.

### Early pathophysiology as a therapeutic window

Across NDDs, the pathophysiological cascade begins well before symptom onset, creating a critical yet underutilized window for intervention. In AD, Aβ accumulation precedes tau aggregation and synaptic dysfunction. In PD, α-synuclein aggregation in the gut and olfactory bulb emerges long before motor symptoms. These observations support the concept of a “silent phase” where intervention could be most effective—if reliable, minimally invasive early detection tools are available.

The convergence of nanotechnology and AI holds promise for capitalizing on this window. Nanobiosensors can detect low-abundance biomarkers, while AI models may stratify risk and predict disease trajectory. However, successful translation depends on more than technological capability; it also requires rigorous clinical validation, user-friendly interfaces, ethical data governance, and health system integration.

### Reframing challenges as design principles

The limitations outlined above—delayed diagnosis, poor biomarker specificity, therapeutic delivery failures, and lack of personalization—are often treated as isolated technical barriers. However, when examined through a translational lens, they can instead be viewed as powerful design imperatives. Each clinical shortcoming presents a clear direction for innovation in AI-integrated nanomedicine.

For example, the chronic delays in diagnosis across NDDs underscore the urgent need for biosensing platforms that are not only ultrasensitive but also scalable and accessible. The ability to detect low-abundance biomarkers in peripheral fluids like blood or saliva should not be a technical luxury but a foundational requirement—especially for systems intended for population-level screening or at-home monitoring.

Similarly, the blood–brain barrier, long considered a therapeutic obstacle, becomes a focal point for nanocarrier engineering. Future delivery systems must account for patient-specific neurovascular heterogeneity and immune responses. This calls for adaptive carriers that can adjust surface chemistry, payload release, or targeting affinity in response to the dynamic brain microenvironment.

And finally, the absence of real-time, personalized treatment monitoring is not merely a clinical gap—it is a blueprint for AI integration. A truly intelligent therapeutic system would not only interpret biosensor signals but also guide drug release, adjust dosing, and learn from longitudinal patient data. Such closed-loop platforms could turn reactive care into proactive, precision medicine.

Ultimately, the path forward lies not in incrementally patching individual limitations but in designing integrated systems where diagnostics, delivery, and decision-making work in concert. By embracing these clinical challenges as design criteria rather than downstream obstacles, the field can move toward technologies that are not only innovative in concept but also viable in the complexity of real-world neuromedicine.

## Biomarker-guided diagnostics and therapeutic strategies

Recent advances in biomarker research have enabled earlier detection and more personalized therapeutic strategies for NDDs. Biomarkers—ranging from Aβ peptides to NfL and phosphorylated tau—offer dynamic insights into disease onset, progression, and therapeutic responsiveness. These molecular signals are not only indicators of pathology but also critical tools for tailoring interventions. When combined with nanotechnology and AI, biomarker-guided strategies have the potential to shift clinical management from reactive to proactive approaches [22].

Unlike cardiovascular or infectious diseases, NDDs suffer from biomarker ambiguity—many indicators like NfL or tau lack disease specificity, appearing in AD, PD, ALS, and MS alike. This overlap complicates differential diagnosis and monitoring but also underscores a shared opportunity: technologies that can measure these biomarkers longitudinally and in real-time could support earlier intervention across all major NDDs.

### Longitudinal monitoring

Tracking biomarkers over time allows clinicians to monitor therapeutic response and disease progression continuously, rather than relying solely on clinical assessments or periodic imaging. This is particularly important in AD and PD, where pathological processes precede clinical symptoms by years or decades [22, 23].

Recent advances in wearable and implantable biosensing devices—often enhanced by AI—have made longitudinal monitoring more feasible outside clinical settings. These platforms now support frequent, minimally invasive sampling (e.g., from sweat, saliva, or interstitial fluid), enabling patients to be monitored in real-world environments. In AD, for instance, wearable EEG sensors combined with plasma p-tau181 tracking can provide correlates of cognitive decline, while in PD, tremor profiles integrated with NfL fluctuations may indicate dopaminergic degeneration [24].

However, scalability remains a challenge. Most AI-enhanced biosensors are still in early-stage validation, and few are approved for routine clinical use. Furthermore, variability in patient adherence, environmental noise, and data interpretation standards remain critical barriers to widespread adoption.

### Preventative screening

The prolonged preclinical window in NDDs presents an opportunity for screening high-risk individuals—provided that biomarkers can be detected early, non-invasively, and cost-effectively. Here, nanosensors capable of detecting femtomolar concentrations of biomarkers in accessible fluids such as blood or saliva are particularly promising [25].

In AD, plasma-based detection of Aβ42/Aβ40 ratios and p-tau181 has demonstrated potential for early diagnosis. In contrast, PD and MS lack similarly well-validated blood-based markers, although interest in exosomal α-synuclein and serum NfL continues to grow [26]. Integration of nanosensor platforms with ML-based signal interpretation reduces false positives and enables stratification based on complex biomarker profiles (Table 1).

Still, current nanosensor prototypes often require specialized fabrication methods, reagent optimization, or microfluidic systems that limit their scalability. Additionally, the regulatory pathway for predictive screening tools remains underdeveloped, especially when AI components are involved.

### Integration with nanomedical platforms

Biomarker data are not only central to diagnosis but are increasingly shaping therapeutic design. Elevated ROS levels in AD or PD can be harnessed by ROS-responsive nanocarriers for targeted drug release, while the overexpression of transport proteins such as RAGE or transferrin receptors at the BBB offers avenues for ligand-mediated nanocarrier targeting to the CNS [27, 28]. In advanced theranostic platforms, real-time biomarker detection can trigger adaptive interventions—whether through controlled drug release, modulation of imaging contrast, or thermally activated responses—creating a feedback loop that aligns therapy with dynamic disease states [29].

Despite these advances, translating multifunctional NPs into clinical practice remains challenging. Many designs lack GMP-compliant production protocols, and the simultaneous validation of diagnostic and therapeutic capabilities is uncommon, complicating regulatory approval, particularly for hybrid AI–nanotechnology systems [30], (Table 1).

**Table 1:** Nano diagnostic platforms for neurodegenerative diseases and multiple sclerosis

Disease	Nanomaterial used	Target biomarker	Detection method	Sample type	Advantages	Ref
Alzheimer’s disease (AD)	Gold nanoparticles (AuNPs)	Amyloid-beta, Tau	Colorimetric/SPR	CSF, Plasma	High sensitivity, simple visualization	[31]
Parkinson’s disease (PD)	Graphene oxide nanosheets	α-synuclein	Electrochemical sensor	Blood, CSF	High conductivity, fast response	[18]
Huntington’s disease (HD)	Magnetic nanoparticles	Huntingtin protein	Magnetic sensing/MRI	Blood	High imaging contrast, non-invasive	[21]
ALS (Amyotrophic Lateral Sclerosis)	Carbon quantum dots	SOD1, TDP-43	Fluorescence-based assay	Serum	High signal-to-noise ratio	[21]
Multiple sclerosis (MS)	Silica nanoparticles	Myelin Basic Protein	Enhanced ELISA	CSF, Serum	Enhanced surface area for better detection	[20]
Alzheimer’s disease (AD)	Microfluidic chips with nanostructures	Tau protein	LSPR biosensor	CSF	Ultra-sensitive early-stage detection	[31]
General NDDs	SERS-active nanoparticles	Neuromodulators/biomarkers	Raman scattering	Tissue/Fluid	High-resolution nanoscale imaging	[25]

Nanotheranostics, as an evolution of nanotechnology-based diagnostics, promise safer and more efficient disease management by addressing the inherent limitations of conventional approaches. Yet, significant barriers remain, including time-intensive NP synthesis, incomplete understanding of nano–biointeractions, and the complexities of CMC required for clinical translation and commercialization. Here, AI—particularly machine learning—offers unique opportunities: automating labor-intensive processes, optimizing NP design, and enhancing predictive modeling for both diagnostic and therapeutic performance [32].

## Nanomedical approaches in neurodegeneration

NDDs share multiple pathological features—chronic inflammation, BBB dysfunction, protein misfolding, and biomarker ambiguity. Nanotechnology provides a unique molecular-scale interface with these pathologies, offering unprecedented capabilities in disease-specific diagnostics, brain-targeted drug delivery, and integrated monitoring systems. However, across AD, PD, and others, the translational maturity of these technologies varies significantly, and their deployment must consider real-world clinical and regulatory constraints [22, 25].

### Nanosensors for early detection

**Fig. 2:**
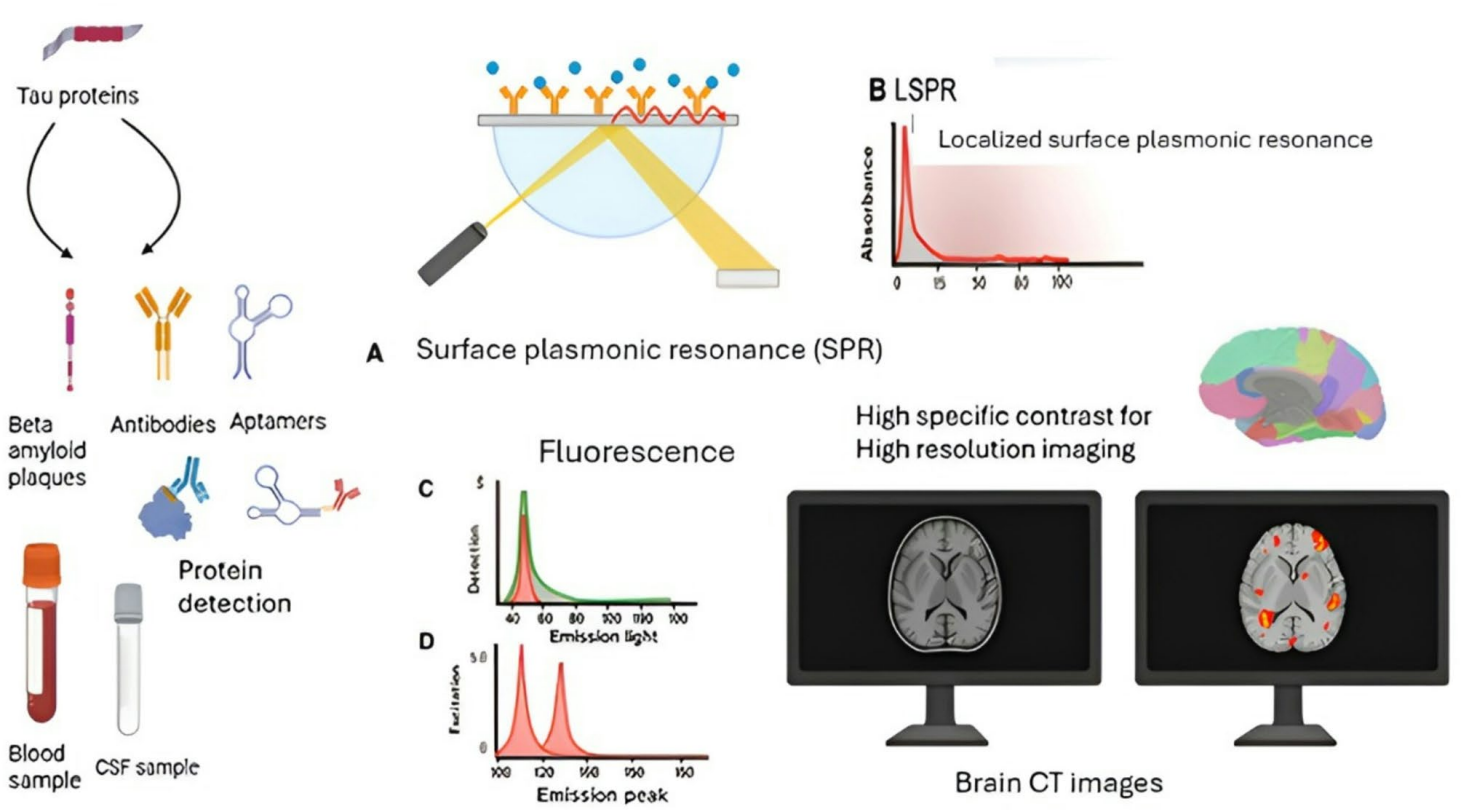
Schematic representation of nanotechnology-enabled strategies for detection and imaging of neurodegenerative disease biomarkers. (**A**) Ex vivo biomarker capture: Tau protein and β-amyloid peptides are selectively recognized by antibodies (Y-shaped symbols) and aptamers (loop structures) from blood and cerebrospinal fluid (CSF) samples. (**B**) Advanced optical detection: Surface-enhanced Raman spectroscopy (SERS), fluorescence spectroscopy, and localized surface plasmon resonance (LSPR) provide sensitive and specific readouts of the captured biomarkers. (**C**) Spectral analysis: Representative fluorescence intensity profiles are shown for β-amyloid signals, illustrating the distinct optical signatures that enable biomarker discrimination. (**D**) In vivo imaging applications: Nanoparticle-assisted MRI and CT imaging improve visualization of β-amyloid plaque deposition in the brain, offering enhanced contrast and diagnostic accuracy. Note: MRI/CT images are schematic illustrations

In diseases such as AD and PD, pathological processes (e.g., Aβ or α-synuclein accumulation) begin long before clinical symptoms. Nanosensors—especially those using gold/silver NPs, graphene, or Quantum dots (QDs) —have enabled detection of low-abundance biomarkers such as Aβ oligomers, phosphorylated tau, and NfL with sensitivities far beyond ELISA-based techniques [23, 25], (Fig. 2).

For instance, SERS biosensors have demonstrated the ability to detect Aβ1–40 at concentrations as low as 100 fg/mL and multiplexed Aβ1–40/1–42 quantification in blood [31, 33]. These advances are particularly significant in AD, where accessible fluid biomarkers are more established. In contrast, reliable blood biomarkers in PD and ALS remain less defined, making sensor specificity more challenging (Table 1).

Despite these advances, most nanosensor platforms remain in the prototype stage. Their integration into routine clinical workflows is hindered by batch-to-batch variability, lack of real-time calibration standards, and the complexity of signal amplification modules—especially for multiplexed detection [34].

### Microfluidics-integrated nano-biosensors

Microfluidic technologies amplify the clinical utility of nanosensors by enabling automated, high-throughput, and low-volume assays (Table 1). These platforms are particularly suited for detecting multiple biomarkers (e.g., Aβ, tau, NfL) in parallel, an essential feature given the overlapping biomarker landscapes across NDDs [35].

In contrast to PD or ALS, where single-biomarker strategies remain limited, AD has benefited from robust multi-analyte panels using microfluidic SERS or LSPR platforms. Such integrated assays also permit genetic profiling—such as APOE genotyping—which can further personalize disease risk models.

However, the manufacturing of these microfluidic-nano hybrids is not yet GMP-compliant in most cases. Fragile components, high cost of fabrication, and complex user interfaces impede their adoption in resource-limited or non-specialist settings. Moreover, validation in large, heterogeneous clinical populations remains a critical missing step [36].

### Nanocarrier systems for targeted drug delivery

Most disease-modifying therapies for NDDs face significant challenges, including limited BBB permeability, off-target effects, and rapid systemic clearance. To overcome these barriers, nanocarriers such as liposomes, dendrimers, PLGA NPs, and polymeric micelles have been engineered with surface modifications, controlled release capabilities, and targeting ligand attachments [27, 37]. For instance, transferrin- and ApoE-conjugated NPs have demonstrated enhanced BBB penetration in AD and PD models, enabling the delivery of antioxidants, siRNA, or neuroprotective agents directly to affected brain regions [28, 38].

Recent advances in AI-driven ligand design, exemplified by the work of David Baker’s group, showcase how computational protein engineering can generate novel, high-affinity targeting moieties. More broadly, AI techniques are increasingly applied in the exploration and optimization of biomedical nanomaterials, accelerating the development of nanocarriers with improved targeting, controlled release, and biocompatibility [39]. These innovations hold promise for improving nanocarrier BBB penetration and enhancing cell-type specificity within the complex neural microenvironment [40]. Complementing this, ROS-responsive carriers exploit the elevated oxidative stress characteristic of AD, PD, and MS to trigger environment-specific drug release, thereby improving therapeutic precision.

A compelling example involves curcumin—recognized for its antioxidant and anti-inflammatory properties—encapsulated within chitosan-coated PLGA NPs. This formulation enhances curcumin’s solubility, stability, and biocompatibility, while enabling controlled release and reducing cytotoxicity. In vivo studies demonstrate that curcumin-loaded NPs achieve superior brain distribution, reduce oxidative stress, and exhibit potential therapeutic effects in AD models [41]. These findings underscore the potential of nanocarrier engineering to significantly boost the efficacy of bioactive compounds.

Despite these promising developments, several challenges remain. Nanoparticle uptake can vary depending on patient-specific BBB receptor expression, and off-target accumulation in organs such as the liver and spleen is common. Furthermore, immune clearance mechanisms can diminish effective dosing, while clinical translation is hindered by issues related to manufacturing scalability, reproducibility in large animal models, and regulatory uncertainties surrounding multifunctional nanocarriers [42].

In parallel, DNA nanocarriers are emerging as a versatile platform for targeted gene therapy. Their intrinsic biocompatibility and programmable structure allow precise loading of nucleic acid drugs via complementary base pairing. For example, sgRNA in sgRNA/Cas9 complexes can be arranged at specific sites on DNA nanocarriers, which can then be functionalized with targeting ligands (small molecules, aptamers, antibodies) and stimuli-responsive elements sensitive to factors such as GSH, pH, or nucleases. Such multifunctional DNA nanocarriers have demonstrated efficient and targeted gene editing capabilities in both in vitro and in vivo models [43].

### Strategies for BBB penetration

**Fig. 3:**
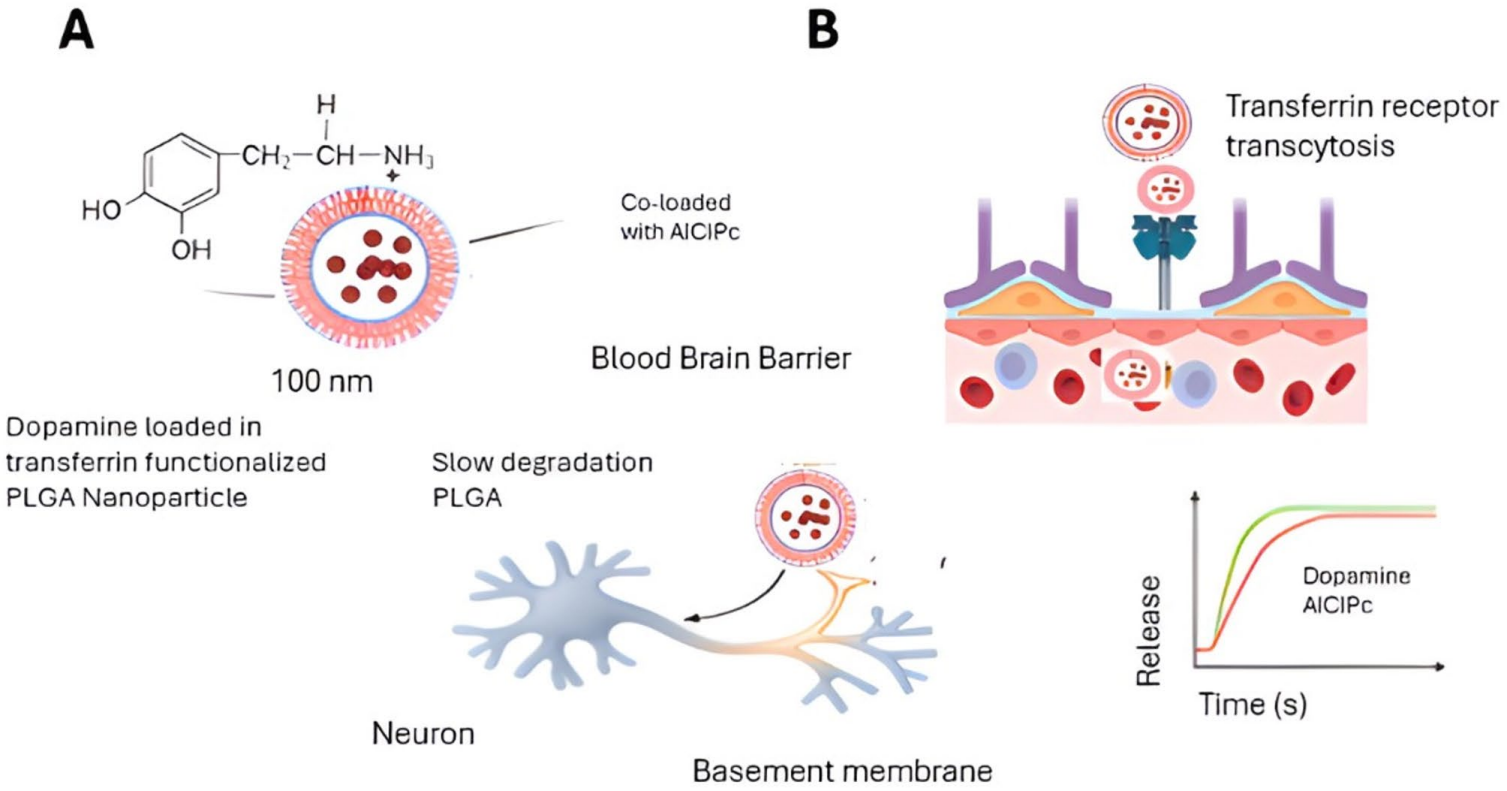
Schematic representation of a dopamine/phthalocyanine-loaded nanosystem for blood–brain barrier transport. (**A**) Nanoparticle structure: Poly(lactic-co-glycolic acid) (PLGA) nanoparticle co-loaded with aluminum chloride phthalocyanine (AlClPc) and dopamine. The schematic illustrates the nanoparticle core and encapsulated therapeutic agents. (**B**) Mechanism of blood–brain barrier (BBB) penetration: The nanosystem traverses brain endothelial cells and subsequently releases dopamine and AlClPc into the brain parenchyma, where they can exert therapeutic effects on neuronal tissue

Given that the BBB serves as both a physical and biochemical gatekeeper, multiple strategies have emerged to facilitate nanocarrier transport into the brain. One of the most widely studied approaches is receptor-mediated transcytosis (RMT), particularly in AD and PD, where ligands targeting transferrin, insulin, or LDL receptors enhance nanoparticle uptake [44]. In contrast, adsorptive-mediated transcytosis (AMT) offers a more general but less selective route, relying on charge-based interactions. While AMT provides broader applicability, it carries an increased risk of cytotoxicity due to non-specific cellular uptake (Fig. 3).

Biomimetic delivery systems have recently gained traction for their ability to traverse the BBB without depending on receptor targeting. Nanoparticles coated with exosomes or cell membranes can evade immune detection and navigate the barrier with greater flexibility—advantages that are especially relevant in diseases like ALS, where receptor targets are often unclear or variable [45, 46]. Complementing these biochemical strategies, physical methods such as focused ultrasound (FUS) combined with microbubbles offer a transient means to open the BBB. However, these techniques remain largely experimental, limited by the necessity for high-precision targeting to minimize off-site effects.

Despite these technical advances, regulatory pathways for BBB-penetrating nanocarriers remain fragmented. Although components such as liposomes have secured FDA approval, the addition of targeting ligands or stimuli-responsive elements introduces new regulatory challenges [47]. Notably, emerging evidence points to the cerebrospinal fluid drainage pathway—mediated by dynamic paracellular pores on venules—as an alternative or complementary route for nanosystem delivery to the CNS. This pathway could potentially bypass some limitations inherent to direct BBB transcytosis, offering a novel avenue for therapeutic delivery [48].

### Theranostic nanoplatforms

Theranostics—integrating diagnostics and therapy within a single nanosystem—represent one of the most ambitious frontiers in nanomedicine. In NDDs, these platforms are being designed to simultaneously deliver drugs, monitor biomarker responses, and report real-time treatment efficacy through imaging modalities such as MRI, PET, or NIR fluorescence [49, 50]. For example, superparamagnetic iron oxide nanoparticles can enable both MRI tracking and magnetically triggered drug release, while NIR-activated platforms can generate localized thermal energy to enhance drug penetration or disrupt pathological protein aggregates.

Recent work has shown that machine learning can accelerate the rational design of such multifunctional theranostic systems, optimizing parameters like biodistribution, target affinity, and stimulus responsiveness to achieve a higher level of integration between diagnostic and therapeutic functions [32].

Despite these advances, the path from bench to bedside remains steep. Theranostic systems are challenging to standardize, present unresolved toxicology concerns, and face bifurcated regulatory pathways—requiring approval as both a therapeutic and a diagnostic device. No theranostic nanoplatform has yet achieved regulatory approval for NDDs, although several are in early-phase clinical trials. Clinical readiness varies widely across technologies, with success hinging on the ability to balance biological innovation with manufacturability, scalability, and clear regulatory frameworks.

Emerging imaging technologies are expanding the potential of these platforms. Near-infrared II (NIR-II) photoacoustic imaging (PAI) offers deep tissue penetration, high spatial resolution, and a high signal-to-noise ratio, making it well suited for early diagnosis, precise localization, and treatment monitoring. When combined with multimodal or diagnostic–therapeutic integrated molecular probes, NIR-II PAI can facilitate accurate disease mapping and guided interventions—not only in oncology but potentially in neurodegenerative applications as well [51]. Recent advances in NIR-II photoacoustic imaging have demonstrated integrated diagnostic–therapeutic systems capable of high-resolution lesion mapping while enabling energy-triggered treatment, underscoring the theranostic potential of this modality in precision neurointervention [51].

## Emerging nanotechnologies

The future of neurodegenerative disease care is being reshaped by a new wave of nanotechnologies that extend far beyond conventional drug carriers or biosensors. These emerging platforms are not only smaller or more sensitive—they are also smarter, more adaptable, and often multifunctional, addressing multiple aspects of disease simultaneously. From advanced imaging to neural repair, they offer novel strategies to detect, modulate, and potentially reverse elements of neural decline.

Among these innovations, NIR-II fluorescent probes have drawn considerable attention for their strong potential in clinical translation. Both inorganic and organic NIR-II probes with excellent imaging performance have recently been developed, combining favorable optical and physicochemical properties with precise targeting capabilities. By integrating nanotechnology with NIR-II imaging, researchers are now creating probes that can selectively accumulate in diseased tissues for both visualization and therapy. These targeting mechanisms may be passive—driven by physiological factors such as the enhanced permeability and retention effect—or active, through the conjugation of ligands that bind disease-specific receptors [52].

### Quantum Dots (QDs): bright probes for early detection

QDs, nanoscale semiconductors known for their brightness and stability, are making strides in brain imaging. Unlike conventional dyes, QDs can be tuned to emit specific wavelengths, allowing for multiplexed detection of different biomarkers in the same tissue sample. When functionalized with peptides or antibodies that bind to disease-specific proteins—like Aβ plaques in Alzheimer’s or α-synuclein in Parkinson’s—QDs can help visualize pathological changes in the brain well before symptoms emerge [53].

However, concerns over long-term toxicity, especially with cadmium-based QDs, remain a major hurdle for clinical translation. Biocompatible alternatives and robust clearance mechanisms are under investigation, but widespread adoption will require extensive safety validation.

### Nanozymes: catalysts for neuroprotection

In diseases where oxidative stress contributes to neuronal injury—such as Alzheimer’s, Parkinson’s, and ALS—nanozymes offer a compelling therapeutic strategy. These NPs mimic the activity of natural enzymes, breaking down reactive oxygen species (ROS) that accumulate in inflamed or damaged brain regions.

Cerium oxide and palladium-based nanozymes, for instance, have demonstrated antioxidant effects and some neuroprotective potential in preclinical models [54]. When incorporated into nanocarriers, these particles may serve dual roles: delivering drugs while also neutralizing harmful oxidative environments. Yet, questions remain about long-term stability, off-target effects, and the consistency of catalytic activity in vivo.

### Magnetic nano-actuators: guiding regeneration

Neuroregeneration remains one of the most elusive goals in treating disorders like ALS or spinal cord injury. Magnetic polymer nano-actuators—engineered NPs that respond to external magnetic fields—are being explored to promote nerve growth, axon alignment, and even synaptic reconnection.

These systems can exert subtle mechanical cues that guide neuronal orientation, potentially aiding tissue regeneration in damaged CNS regions [55]. While this is an exciting frontier, most applications are still in early animal models, and it’s unclear how well such physical interventions will translate to complex human neural environments.

### Nanophotonics and chiral metamaterials: precision biosensing

Some of the most advanced biosensing platforms now draw on chiral metamaterials—engineered structures that can detect changes in protein shape based on how they twist light. This is especially relevant in NDDs, where the misfolding of proteins like tau, α-synuclein, or TDP-43 defines pathology.

Unlike conventional biomarker assays, chiral biosensors aim to detect not just the presence but the conformation of these proteins, allowing for ultra-early detection with minimal invasiveness [56]. However, manufacturing such sensors at clinical scale remains complex and costly. Their future depends on miniaturization, automation, and integration into broader diagnostic systems.

### Hybrid nanoplatforms: the best of both worlds

To overcome the limitations of single-material systems, researchers are now combining organic and inorganic nanomaterials—like gold-iron oxide composites or polymer-metal hybrids—into multifunctional platforms. These hybrids can simultaneously act as imaging agents, drug carriers, and biosensors.

For example, a gold core might provide optical signal enhancement, while an iron oxide shell allows for MRI tracking, and the surface could be coated with therapeutic or targeting ligands [57]. The versatility is promising, but integrating these diverse functions without compromising safety, stability, or manufacturing scalability is a significant challenge.

While these emerging nanotechnologies offer exciting potential, the path to clinical application is far from straightforward. Many platforms show strong preclinical promise, but hurdles such as manufacturing reproducibility, regulatory approval, immunogenicity, and in vivo variability must be addressed head-on. In particular, multifunctional systems that combine imaging and therapy will face complex approval processes, requiring demonstration of both safety and efficacy in multiple functional domains.

The next stage of progress will depend not only on technical innovation but also on interdisciplinary collaboration—among neuroscientists, materials engineers, clinicians, and regulatory bodies—to ensure that these technologies are robust, scalable, and truly responsive to patient needs.

## The role of artificial intelligence in neurodegenerative disease management

AI is no longer a futuristic tool in the study of NDDs—it is now an essential ally. Its growing role spans early diagnosis, biomarker analysis, patient stratification, and even therapeutic decision-making. Yet, while its contributions are increasingly impactful, the real test lies in aligning algorithmic sophistication with the clinical realities of AD, PD, ALS, and other complex neurological conditions.

### Imaging analysis: from visual assessment to precision mapping

Traditionally, radiological assessment of neurodegenerative changes has relied on expert interpretation of MRI, CT, or PET scans—an approach inherently limited by interobserver variability and subtle, early-stage features that are difficult to detect. AI, particularly machine learning (ML) and deep learning (DL) models, has significantly improved the sensitivity and specificity of image-based diagnostics. These systems can automatically detect brain atrophy, cortical thinning, white matter hyperintensities, and patterns of hypometabolism long before clinical symptoms become apparent [58].

For example, in AD, DL-based MRI classifiers have been shown to outperform conventional volumetric techniques in identifying hippocampal atrophy. In PD, AI can enhance the detection of nigrostriatal degeneration, even at prodromal stages. However, these tools face translation barriers including data heterogeneity across imaging centers, lack of standardized imaging protocols, and challenges in regulatory approval for clinical-grade diagnostic AI systems [59].

### Predictive modeling: capturing disease trajectories

Beyond static snapshots, AI excels at modeling longitudinal disease trajectories using multimodal data—including genomics, proteomics, electronic health records, and sensor-derived inputs. These models can stratify patients into molecular or progression subtypes, enabling more precise prognosis and tailored interventions. For instance, AI has been used to predict conversion from mild cognitive impairment (MCI) to AD with over 80% accuracy in select datasets [60].

Yet, a key limitation is that many models are developed on highly curated, single-institution cohorts that do not reflect the variability of real-world populations. This raises concerns about algorithmic bias, overfitting, and reduced generalizability to diverse clinical settings. Rigorous external validation and regulatory guidance on clinical-grade model deployment remain urgently needed.

### Drug discovery acceleration

The time and cost associated with traditional drug development—often exceeding 10 years and billions of dollars—pose major obstacles in NDD research. AI-driven platforms now assist in multiple stages: virtual screening, compound de novo design, and drug-target interaction prediction. These approaches have identified repurposable drugs and novel leads for protein misfolding diseases, such as tauopathies and synucleinopathies [61].

However, while AI reduces early-phase discovery timelines, its success still hinges on robust experimental validation. Predicted hits often fail due to poor pharmacokinetics or toxicity profiles—limitations that AI alone cannot circumvent. Furthermore, the integration of AI outputs into regulatory pathways for drug approval is still evolving and lacks consensus frameworks for validation standards.

### Real-time monitoring and adaptive care

Wearable devices and digital biomarkers are increasingly common in NDD trials and clinical practice. AI systems interpret this continuous data stream—tracking gait, tremor frequency, voice changes, sleep disturbances, and even subtle shifts in facial expression. In PD, such tools have been used to detect off-periods and optimize dopaminergic therapy. In ALS, AI-supported voice analysis can capture bulbar deterioration earlier than clinical assessments [62].

These capabilities enable real-time, patient-specific adjustments to care. But device calibration, user compliance, and data privacy concerns must be addressed before wide adoption. Moreover, despite promising results, very few wearable-AI systems have received regulatory clearance for clinical decision support.

### Toward closed-loop systems: synergy with nanotechnology

Perhaps the most transformative potential lies in closed-loop therapeutic systems, where AI interprets nanosensor outputs (e.g., inflammatory markers or protein aggregates) and directs responsive nanocarriers to adjust drug release accordingly. This real-time feedback architecture bridges diagnostics and therapy—moving from reactive care to proactive intervention [63].

However, such integration is still conceptual. Challenges include synchronizing sensor readouts with therapeutic actuation, managing latency in data interpretation, and ensuring biocompatibility of sensor-delivery combinations. Moreover, regulatory pathways for dynamic, AI-controlled therapeutics are undeveloped, creating uncertainty for clinical translation.

### Critical perspective: AI as a co-pilot, not a silver bullet

AI is increasingly positioned as a transformative force in the diagnosis and treatment of NDDs. Its ability to integrate diverse data modalities—ranging from imaging and genomics to wearable sensor outputs—offers unprecedented opportunities to decode complex disease processes and personalize care. When paired with the molecular precision of nanotechnologies, AI can help design targeted interventions, optimize therapeutic timing, and predict disease trajectories more accurately than ever before.

However, this promise must be tempered with realism. Most AI tools remain under-validated in real-world clinical settings, with limited interoperability across healthcare systems. A critical barrier lies in the lack of harmonized, representative datasets across institutions, which increases the risk of algorithmic bias and reduces generalizability. Moreover, many models still function as opaque “black boxes,” making it difficult for clinicians to interpret results with confidence—an issue that directly impacts trust and adoption.

To move from hype to utility, future work must emphasize transparency and explainability, especially in high-stakes applications. The development of regulatory frameworks that specifically address the convergence of AI and nanomedicine is also essential to guide ethical deployment and streamline approval.

Ultimately, AI is unlikely to replace clinicians or biomedical researchers. Instead, its greatest value lies in its role as a co-pilot—augmenting human expertise with speed, scalability, and analytical depth. When thoughtfully integrated, AI can sharpen clinical insight, accelerate discovery, and support more adaptive, responsive care strategies.

## AI in imaging analysis

Neuroimaging plays a pivotal role in the diagnosis and monitoring of NDDs, but conventional methods often fall short in early detection due to limited sensitivity and subjectivity. AI, particularly DL and convolutional neural networks (CNNs), is reshaping this landscape by enabling automated, sensitive, and reproducible analysis of structural and molecular imaging data (Table 2).

**Table 2:** Recent applications of artificial intelligence (AI) in neurodegenerative disease research

Application	Description	AI technique used	ND focus	Ref
Neuroimaging analysis	Detection of hippocampal atrophy, cortical thinning, and DTI-based white matter degeneration	Deep learning (CNNs)	Alzheimer’s, Parkinson’s	[1, 2]
Disease progression modeling	Forecasting conversion of MCI to dementia, motor decline, ALS survival	Supervised ML, XAI	Alzheimer’s, Parkinson’s, ALS	[3, 4]
Drug discovery & repurposing	Virtual screening, generation of novel neuroprotective molecules	GANs, GNNs, Deep RL	Alzheimer’s, Parkinson’s	[5]
Multi-omics integration	Correlating genomics, proteomics, and metabolomics for biomarker discovery	t-SNE, UMAP, clustering, semi-supervised learning	Alzheimer’s, ALS, MS	[6]
Wearable neurotechnology	Monitoring gait, tremors, EEG signals for early detection and severity tracking	AI-enhanced signal processing	Alzheimer’s, Parkinson’s	[7, 8]
AI-guided nanoparticle design	Predicting BBB permeability, ligand density optimization	Supervised ML, Bayesian optimization, GANs	Glioblastoma, Alzheimer’s	[9]
Smart nanodevices	Real-time biosensing and AI-triggered drug release based on biomarkers	AI pattern recognition	Alzheimer’s, Parkinson’s	[10]
Closed-loop neurofeedback systems	EEG-based feedback for deep brain stimulation and drug release	Reinforcement learning, adaptive control	Parkinson’s, Alzheimer’s	[10]
Digital twin modeling	Simulation of brain degeneration, in silico testing of nanotherapies	AI-based physiological modeling	Alzheimer’s, Parkinson’s	[12]

In AD, AI models have demonstrated superior performance in identifying early markers such as hippocampal atrophy and cortical thinning—features often missed in manual reads. In PD, DL applied to diffusion tensor imaging (DTI) supports the quantification of white matter integrity and substantia nigra degeneration, offering new, non-invasive biomarkers of disease burden. Unlike AD, where cortical biomarkers dominate, PD imaging benefits from AI’s capacity to detect subcortical microstructural changes [64].

These tools also facilitate the integration of multimodal imaging data. For example, AI has been used to combine MRI with PET to map amyloid or tau burden, improving diagnostic precision and enabling longitudinal tracking in preclinical and early-stage AD [65]. In contrast, AI-based lesion tracking in MS—a chronic neuroinflammatory disease with neurodegenerative components—emphasizes spatial and temporal lesion dynamics, highlighting how disease-specific priorities shape AI applications across neurological disorders [66].

Despite promising results, translational hurdles remain. Many models are trained on homogeneous datasets, limiting generalizability. Standardization of imaging protocols, external validation, and regulatory clarity are essential for clinical adoption. Furthermore, explainability remains a key challenge, particularly in high-stakes diagnostic decisions.

**Fig. 4:**
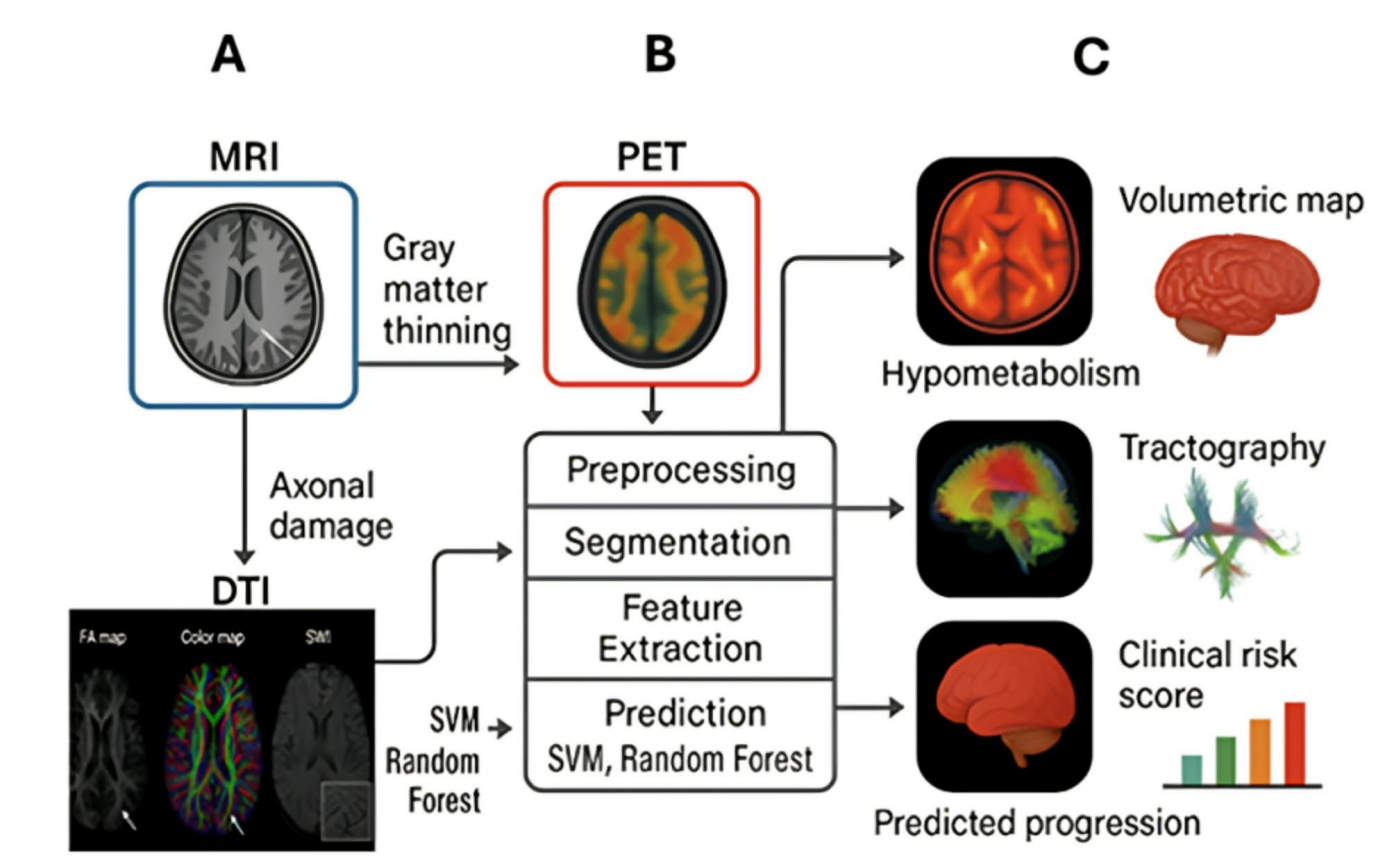
Schematic integration of artificial intelligence (AI) with neuroimaging for monitoring neurodegeneration. (**A**) Pathological features: Key imaging markers include hippocampal atrophy, white matter disruption, and amyloid-β plaque accumulation. (**B**) AI pipeline: Multi-step workflow involving preprocessing, brain region segmentation (convolutional neural networks), feature extraction (e.g., cortical thickness, PET standardized uptake value ratio [SUVR] maps, diffusion tensor imaging [DTI] fractional anisotropy), and disease risk prediction using deep learning models (e.g., ResNet, XGBoost). (**C**) Clinical outputs: Schematic examples include heatmaps of cortical atrophy, tractography of white matter pathways, and predictive dashboards integrating imaging features with cognitive scores and cerebrospinal fluid (CSF) biomarkers

AI’s role in imaging is not to replace radiologists but to augment clinical decision-making with greater precision, speed, and objectivity. As these tools mature, their integration with nanoscale diagnostics and biomarker monitoring may support closed-loop systems for real-time disease management (Fig. 4).

## AI-driven strategies in neurodegenerative disease management

AI is increasingly positioned as a critical enabler in the fight against NDDs. By navigating the high-dimensional complexity of NDD biology—spanning multi-omics data, neuroimaging, behavioral phenotypes, and real-world signals—AI holds potential to address bottlenecks in prediction, treatment optimization, and monitoring. In this section, we explore four core domains where AI is reshaping the landscape of neurodegenerative care: disease progression modeling, drug discovery, multi-omics integration, and wearable neurotechnology.

### Predictive modeling of disease progression

One of AI’s most compelling contributions to neurology is its ability to forecast disease trajectories at the individual level. In AD, ML models trained on multimodal data—including neuroimaging, cognitive tests, and genetic markers—can predict the likelihood and timing of conversion from MCI to dementia [67]. Similarly, in PD, AI tools are used to model future motor decline, while in ALS, predictive algorithms assess respiratory function deterioration and overall survival.

These models support clinicians in anticipating care needs and adjusting interventions early, before irreversible loss occurs. Importantly, they also improve patient stratification in clinical trials, enabling more sensitive detection of therapeutic effects and reducing cohort heterogeneity. The emergence of explainable AI (XAI) tools adds a layer of transparency, helping clinicians interpret predictions through visual explanations and interpretable risk scores—critical for clinical adoption and trust.

Yet, real-world application remains limited by variability in training datasets, lack of standardization across centers, and the need for prospective validation before regulatory integration.

### AI-driven drug discovery platforms

Traditional drug development for NDDs is notoriously slow, high-risk, and costly—further complicated by the blood–brain barrier (BBB), complex pathophysiology, and the limited predictive power of animal models. Here, AI offers a more efficient alternative by transforming how candidate drugs are identified and prioritized.

DL algorithms—including generative adversarial networks (GANs), graph neural networks (GNNs), and deep reinforcement learning—are being used to screen virtual compound libraries, model drug–target interactions, and optimize lead candidates. This includes screening for modulators of β-secretase (BACE1), tau kinases, or α-synuclein aggregation pathways, all critical targets in AD and PD [68].

AI also facilitates drug repurposing by mining biomedical literature and real-world datasets to uncover existing compounds with unexplored neuroprotective or anti-inflammatory properties. Moreover, predictive modeling of BBB permeability, toxicity, and metabolism allows early triage of non-viable compounds—reducing late-stage failure.

While these platforms are powerful, the leap from virtual predictions to clinical translation hinges on reliable wet-lab validation, GMP-compliant synthesis, and regulatory clarity on AI-guided therapeutic pipelines.

### Multi-omics integration and subtyping

The multifactorial nature of NDDs—spanning genetics, epigenetics, proteomics, and metabolomics—demands analytical frameworks capable of capturing non-linear interactions across biological systems. AI, particularly unsupervised and semi-supervised learning, provides tools to integrate and interpret such high-dimensional datasets.

Clustering algorithms (e.g., k-means, hierarchical clustering) and dimensionality reduction techniques (e.g., t-SNE, UMAP) are helping researchers identify disease subtypes and stratify patients based on molecular signatures. In AD, for instance, AI has revealed novel networks linking risk genes (e.g., APOE4, BIN1) to dysregulated immune responses and impaired synaptic function [69]. Similar frameworks in ALS and MS (primarily an inflammatory disorder with neurodegenerative aspects) have uncovered pathologically distinct patient subsets, offering new avenues for targeted therapy.

However, while integration across omics layers offers exciting insight into NDD mechanisms, reproducibility remains a concern due to cohort-specific biases, limited longitudinal datasets, and disparities in data preprocessing. Translating these insights into actionable diagnostic panels or targeted interventions will require harmonization across biobanks and prospective multi-omics trials.

### AI in wearable neurotechnology

As NDD management increasingly shifts toward decentralized and patient-centric care, wearable neurotechnology emerges as a powerful ally. Devices like smartwatches, EEG headbands, and inertial measurement units (IMUs) now continuously capture behavioral, motor, and physiological data from patients in their everyday environments.

AI algorithms analyze these data streams to detect early deviations from baseline, such as reduced gait velocity in PD or altered sleep-wake cycles in AD. For example, DL applied to EEG signals has shown potential in detecting early cognitive changes in preclinical AD, while AI-enhanced IMUs quantify bradykinesia or tremor with greater precision than clinical scales [54].

These systems not only support early intervention but also generate granular endpoints for clinical trials, reducing reliance on subjective clinician assessments. They are particularly impactful in low-resource or rural settings, expanding access to specialist care.

That said, challenges remain around data privacy, algorithm bias, device standardization, and user compliance. The long-term success of AI-powered wearable platforms will depend on seamless integration with electronic health records and robust data governance frameworks.

Together, these AI-driven strategies offer a multi-pronged approach to modernizing neurodegenerative disease management—from early prediction and personalized treatment to data-driven monitoring and accelerated drug pipelines. Yet, realizing their full potential requires addressing systemic barriers: dataset diversity, model interpretability, cross-site reproducibility, and regulatory pathways for digital tools. Future progress will depend on interdisciplinary collaboration, co-development with clinicians, and a translational mindset that bridges innovation with clinical realities.

## Synergistic integration of nanotechnology and AI in neurodegenerative care

The convergence of nanotechnology and AI is ushering in a new generation of intelligent systems for the diagnosis, monitoring, and treatment of NDDs. Each field offers distinct advantages—nanomedicine excels at molecular precision, while AI thrives on data-driven adaptability. Together, they promise dynamic platforms capable of responding to real-time pathophysiological changes and personalizing interventions with unprecedented granularity.

What distinguishes this integration is its potential to overcome longstanding bottlenecks in translational neuroscience: the heterogeneity of NDD progression, the opacity of treatment response, and the lack of continuous, patient-specific feedback. However, while conceptually compelling, these systems remain largely in the preclinical stage. Their path to clinical relevance hinges not just on innovation but also on validation, regulatory pathways, and scalability.

### AI-guided nanoparticle design

Designing NPs for targeted CNS delivery is a multidimensional challenge involving trade-offs among size, surface chemistry, ligand density, biostability, and BBB permeability. Traditionally, optimizing these parameters relied on time-consuming trial-and-error experimentation. AI now offers a powerful alternative.

ML models, trained on physicochemical descriptors and biological outcomes, can predict how specific NP configurations affect BBB penetration, cellular uptake, or clearance. Reinforcement learning algorithms iteratively adjust design features to maximize therapeutic payload delivery while minimizing toxicity or immunogenicity [70].

Generative models such as variational autoencoders and GANs are being used to propose novel nanoparticle architectures suited to disease-specific conditions—e.g., redox-sensitive NPs for oxidative microenvironments in AD or inflammatory-targeting carriers for MS lesions.

One particularly promising example is a lab-in-the-loop system that combined ML with high-throughput synthesis and in vivo testing to optimize PEGylation levels, ligand ratios, and NP morphology. This framework, validated in glioblastoma models, significantly improved brain accumulation and therapeutic response [71]. While early data are encouraging, most platforms are yet to be validated in neurodegenerative disease contexts, and their generalizability remains uncertain.

### Smart nanodevices with real-time AI-based monitoring

Passive drug carriers are giving way to intelligent nanosystems capable of sensing disease biomarkers and autonomously triggering therapeutic responses. These “smart” nanodevices incorporate biosensors and AI modules that monitor local neurochemical states—such as pH, ROS, or neurotransmitter levels—and adjust therapy in real time.

In AD, for instance, a rise in Aβ oligomers in CSF could trigger local drug release via redox-sensitive nanocarriers. AI algorithms enable these systems to identify temporal biomarker patterns, distinguish noise from signal, and optimize release kinetics [70].

This concept holds particular promise in diseases with fluctuating symptom severity, such as PD or relapsing MS, where real-time intervention could substantially improve quality of life. However, engineering constraints around power consumption, data transmission, and biosafety of nanodevice implantation remain unsolved. Moreover, regulatory frameworks for autonomous therapeutic systems are still emerging, especially in chronic CNS conditions.

### Closed-loop neurotherapeutics

The vision of closed-loop systems—where sensing, computation, and actuation occur in continuous feedback—lies at the core of precision neuromedicine. By integrating AI-guided analytics with nanomedical payloads or neuromodulatory devices, these systems offer dynamic, personalized responses to evolving disease states.

For example, smart deep brain stimulation (DBS) implants in PD can now use local field potentials to modulate stimulation parameters in real time, improving motor control while reducing cognitive side effects. A future step is integrating NP-based drug reservoirs within such platforms, enabling concurrent chemical and electrical modulation based on AI interpretation of biosignals [71].

**Fig. 5:**
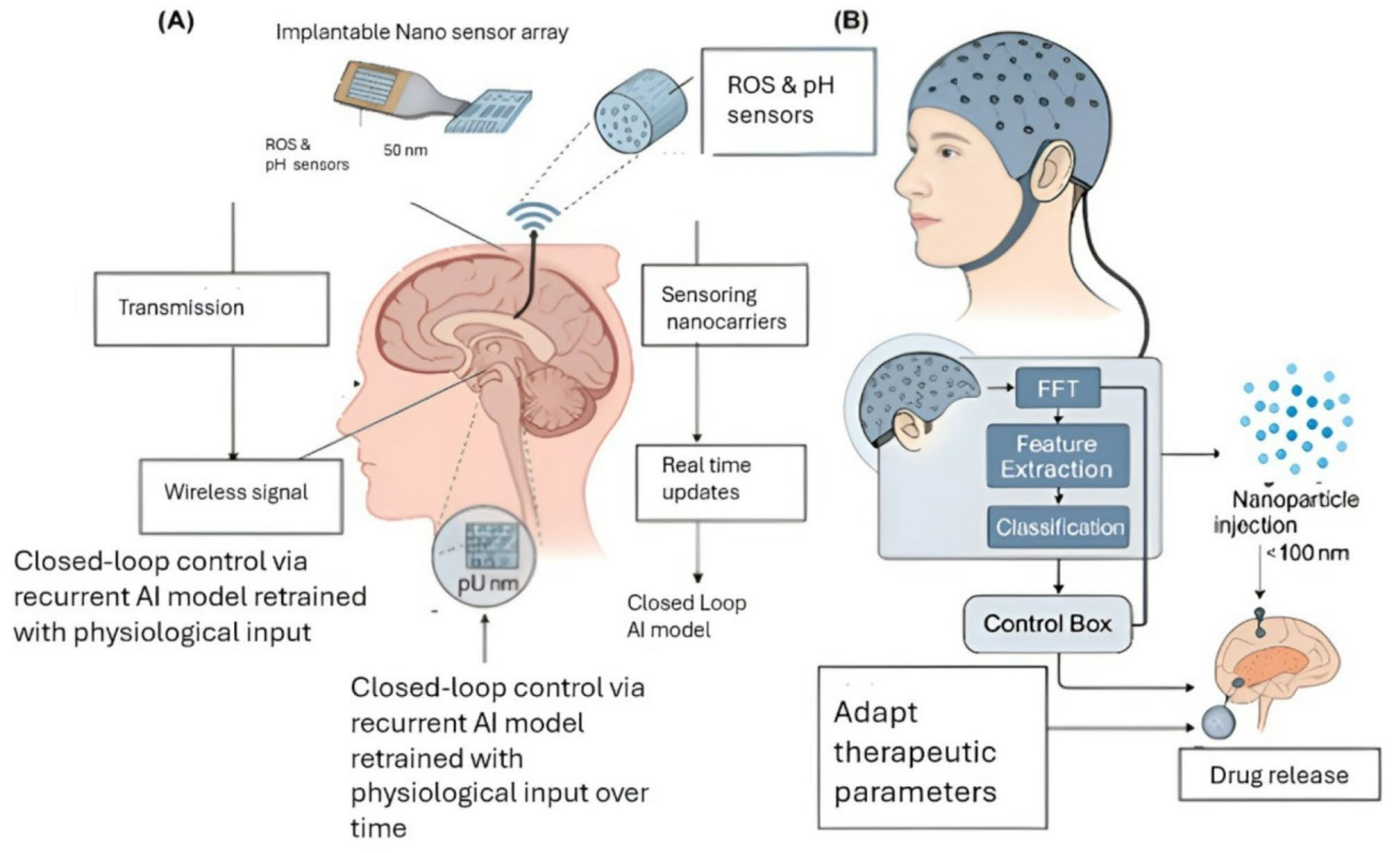
Schematic illustration of AI-integrated smart nanodevices and closed-loop neurotherapeutic systems. A) The left panel shows implantable nanoelectronic sensors designed for real-time neurochemical monitoring. These devices detect biomarkers such as land reactive oxygen species (ROS) and transmit the data to AI systems for analysis. Upon identification of pathological signals, responsive nanocarriers release therapeutic agents in a targeted manner, providing feedback-driven treatment with minimal off-target effects. B) The right panel depicts a closed-loop therapeutic platform that integrates nanoparticle-based drug delivery, AI-mediated signal processing, and electroencephalogram (EEG) signal acquisition. This adaptive system adjusts therapeutic interventions in real time based on neurophysiological feedback, representing a potential pathway toward personalized, self-regulating neurotherapeutics

In AD, early-stage work has explored EEG-triggered NP release systems, where shifts in theta or beta wave activity serve as proxies for cognitive load and therapeutic need. These interventions represent a fundamental shift—from static dosing schedules to real-time, adaptive therapy (Fig. 5).

Despite conceptual breakthroughs, these platforms are in nascent stages. Long-term stability, patient safety, and real-world data integration remain significant hurdles to clinical deployment. Clinical-grade versions will require coordinated input from neurologists, materials scientists, regulators, and ethicists.

### Digital twin models of neurodegeneration

Digital twin models—dynamic, computational replicas of individual patients—represent a culmination of the AI–nanomedicine interface. By integrating multimodal data from wearable sensors, nanoscale diagnostics, and clinical records, digital twins can simulate disease progression and predict patient-specific responses to interventions.

**Fig. 6:**
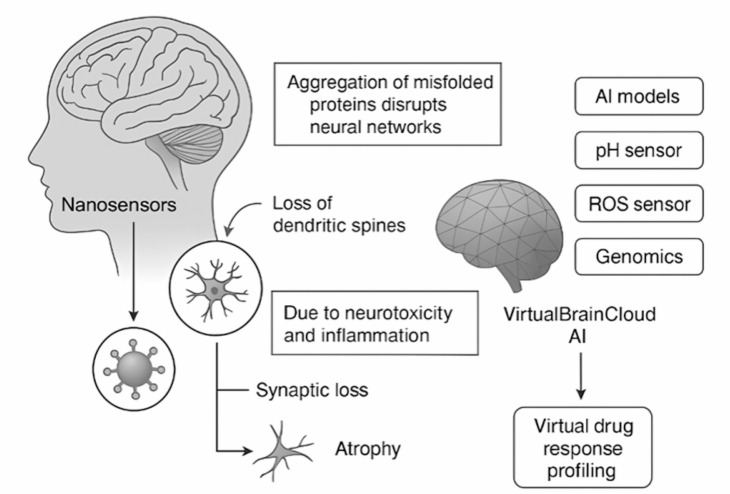
Schematic representation of digital twin modeling for neurodegenerative disease progression. A patient-specific digital twin of the brain is constructed by integrating biomarker profiles, nanosensor readouts, and clinical imaging data through artificial intelligence (AI) models. The virtual model simulates key pathological processes—including protein aggregation, synaptic dysfunction, and neuronal atrophy—in a temporally coherent manner. This platform enables in silico testing of therapeutic strategies, such as nanoparticle-based drug delivery, to optimize treatment timing and dosage, ultimately supporting personalized intervention strategies

In theory, such models could track Aβ aggregation in virtual hippocampal regions, simulate drug diffusion from NPs through brain parenchyma, and adapt therapeutic strategies in silico before real-world application. Over time, these systems could serve as personal decision-support engines, guiding clinicians in treatment selection, dosing, and timing [72], **(**Fig. 6**)**.

Realizing this vision requires substantial infrastructure—interoperable data streams, longitudinal biomarker monitoring, regulatory frameworks for in silico testing, and clinician-facing interfaces that support rather than overwhelm decision-making. While digital twins are already gaining traction in oncology and cardiology, their application in NDDs remains largely theoretical. Progress here will likely be incremental, moving from population-level simulations to semi-personalized models before achieving full digital individuality [73].

The integration of AI and nanomedicine represents more than a technological advance—it’s a reimagining of how we understand, monitor, and treat neurodegenerative disorders. These synergistic systems have the potential to convert episodic, reactive care into dynamic, continuous precision management.

Still, clinical translation will not hinge on innovation alone. Real-world success demands attention to manufacturing reproducibility, patient usability, regulatory alignment, and ethical design. The next decade will test whether these promising technologies can move from proof-of-concept to patient bedside—and whether they can do so equitably, ethically, and effectively.

## Challenges and ethical considerations in nano-AI neurotechnologies

As nanotechnology and AI converge to reshape the landscape of NDD care, their promise is matched by a complex set of challenges. Scientific limitations, ethical dilemmas, regulatory uncertainty, and questions of equity and access form a multifaceted barrier to clinical translation. Addressing these issues head-on is not peripheral—it is central to realizing meaningful, trustworthy innovation in neurology.

### Nanotoxicity and long-term safety

While nanomedicine offers unparalleled precision in delivery and sensing, concerns about nanotoxicity persist—especially in the context of chronic diseases like AD or PD that may require long-term or repeated dosing. Due to their high surface area-to-volume ratios, NPs can generate oxidative stress, disrupt cell membranes, or accumulate in non-target tissues, leading to unintended cytotoxic or immunogenic responses.

A striking example was the 2024 suspension of a ceria-based NP trial in Alzheimer’s due to neuroinflammatory responses in murine models—despite promising in vitro results. This underscores the gap between lab-based performance and in vivo behavior.

Additionally, questions about biodegradability, excretion pathways, and long-term bioaccumulation remain largely unanswered for many advanced nanomaterials. Unlike traditional small-molecule drugs, there are few standardized frameworks for evaluating chronic exposure effects of nanomaterials on the CNS or peripheral systems.

Robust safety pipelines will require physiologically relevant platforms—including BBB-on-chip models, brain organoids, and longitudinal in vivo imaging—to simulate and track NP behavior in complex neural environments. Without these tools, regulatory and ethical approval for chronic NDD applications will remain elusive.

### AI transparency, bias, and data privacy

AI’s ability to process multimodal neurological data offers clear benefits—but it comes with its own risks. Many DL models operate as opaque “black boxes,” making it difficult for clinicians to interpret or trust their outputs, especially in high-stakes scenarios such as differential dementia diagnosis.

Moreover, AI systems trained on demographically skewed datasets may produce biased predictions, reinforcing existing health inequities. For example, several dementia progression models have shown reduced accuracy for underrepresented ethnic groups or low-resource clinical environments. Small-scale, single-center biological datasets often limit reproducibility and external validation, underscoring the necessity of multi-site, standardized data collection to enhance the robustness of AI models and nanomedicine translational studies.

XAI techniques—such as SHAP, LIME, and attention mapping—are increasingly being used to improve interpretability. However, interpretability alone does not resolve the problem of data quality. Diverse cohort representation, inclusive data sourcing, and bias audits during model development must become the norm, not the exception.

Meanwhile, the use of sensitive neurodata (e.g., brain imaging, EEG, genetic information) raises fundamental questions of ownership, consent, and cybersecurity. AI-driven platforms must implement privacy-preserving strategies such as federated learning and differential privacy to align with international standards like GDPR and HIPAA—especially when integrated with cloud platforms or wearable devices.

### Regulatory gaps for hybrid systems

Nano-AI technologies defy traditional regulatory categories. Are they drugs, devices, software, or all three? Most existing frameworks—including the FDA’s Software as a Medical Device or EMA’s advanced therapy guidelines—were not designed for multifunctional, adaptive platforms that combine intelligent sensing, diagnostics, and therapeutic delivery.

These ambiguities create bottlenecks for approval. Regulatory “sandboxes”—such as those piloted by the UK MHRA and Singapore HSA—offer promising models for iterative, real-world testing under adaptive oversight. Still, harmonized global standards will be essential to streamline development for diseases like AD, where international trial coordination is often necessary.

Moreover, real-time updating systems (e.g., AI platforms that evolve with incoming data) challenge the very idea of static product approval. Future pathways must address how to validate continuously learning systems, ensure safety under data drift, and define the limits of post-market modification.

### Economic and equity considerations

While the convergence of AI and nanomedicine holds the potential to accelerate progress toward personalized neurology, it also presents a critical risk: deepening the digital and economic divide in healthcare. The development and deployment of these advanced systems—whether through the manufacture of complex nanocarriers, the training of large AI models, or the maintenance of secure data infrastructures—are often resource-intensive. For many low- and middle-income countries, such technologies may remain inaccessible, potentially exacerbating global disparities in neurodegenerative disease management.

To ensure that innovation does not come at the expense of equity, future efforts must prioritize accessibility and scalability from the outset. Promising directions include the use of paper-based nanosensors analyzed through cloud-based AI platforms, edge AI models that operate on low-power portable devices without relying on continuous internet connectivity, and open-source diagnostic algorithms that can be adapted to the genetic, environmental, and clinical contexts of local populations.

Achieving equitable implementation will also require sustained collaboration across sectors. Public health institutions, private technology developers, and global health organizations must come together to fund, scale, and distribute these solutions in a way that ensures they reach the communities most in need—not just those with the resources to afford them.

### Toward ethical and sustainable implementation

Ultimately, the challenge is not simply to innovate, but to do so ethically, inclusively, and sustainably. Ethical frameworks grounded in beneficence, autonomy, non-maleficence, and justice must guide every stage of nano-AI development—from data collection and model design to clinical deployment.

Ongoing stakeholder engagement—across patients, caregivers, ethicists, clinicians, and developers—is essential to align these tools with lived realities, avoid techno-solutionism, and build enduring trust.

The next phase of neurodegenerative care will not be driven by technology alone, but by the values embedded in that technology. With foresight, inclusivity, and rigor, the convergence of AI and nanomedicine has the potential to catalyze not just smarter tools—but a more compassionate and equitable model of precision neurology.

## Future directions and opportunities in nano-AI neurotechnologies

The dual crisis of rising NDD burden and inadequate therapeutic options has created an urgent need for paradigm-shifting solutions. The convergence of nanotechnology and AI holds extraordinary promise to reshape the trajectory of neurodegenerative care—from late-stage symptom management toward predictive, preventive, and precision strategies. Yet the path forward is neither linear nor guaranteed. Meaningful progress will depend on cross-sector collaboration, translational pragmatism, and ethical foresight.

### Personalized nanomedicine guided by AI

Current NDD therapies remain largely one-size-fits-all, despite well-known interpatient variability in genetics, biomarker expression, and treatment response. The next frontier is personalized nanomedicine, where AI integrates longitudinal clinical data, imaging biomarkers, and multi-omics profiles to tailor interventions at the molecular level.

Advanced ML models can now simulate disease trajectories and therapeutic responses in silico, helping optimize nanoparticle formulations, payload types, and dosing regimens for individual patients. For instance, AI may suggest ROS-responsive drug delivery systems in patients with high oxidative stress signatures, or modify BBB-targeting ligands in response to evolving neurovascular profiles.

The ability to update treatment plans in real time—based on continuous input from biosensors or wearable neurotech—promises a dynamic, patient-specific care paradigm. However, such systems also raise substantial regulatory and reimbursement challenges. Clinical integration will require not only predictive accuracy but also explainability, safety validation, and seamless interoperability with health records.

### Hybrid biosensors and smart neural implants

The shift from episodic, clinic-based assessments to continuous physiological monitoring is another transformative trend. Hybrid biosensors—built from graphene, QDs, or plasmonic substrates—can detect nuanced neurochemical or electrophysiological signals, including pH shifts, inflammatory cytokines, or neurotransmitter flux.

When embedded in intelligent implants and paired with AI processors, these sensors become part of closed-loop systems capable of both detecting pathological changes and triggering therapeutic actions. In PD, for example, smart deep brain stimulation devices can modulate stimulation parameters in real time based on local neural oscillations. In AD, implantable nanosensors may detect surges in Aβ oligomers and initiate targeted drug release before cognitive decline becomes clinically apparent.

Although early prototypes show promise, long-term biocompatibility, power supply constraints, and surgical risks remain barriers. Large-scale deployment will also require breakthroughs in materials science, fail-safe design, and cross-disciplinary surgical expertise.

### Cross-disciplinary collaboration and innovation ecosystems

Innovation at the intersection of nanotech and AI cannot succeed in disciplinary silos. The development of effective, safe, and accessible neurotechnologies demands deep collaboration among neuroscientists, materials chemists, AI engineers, clinicians, regulators, ethicists, and—critically—patients themselves.

Creating translational innovation ecosystems—such as academic-industry consortia, open-data platforms, and real-world testing networks—can help accelerate product development while ensuring societal relevance. Education and training programs that bridge neuroscience, computation, and engineering will be essential to cultivating a workforce fluent in convergent biotechnologies.

Importantly, co-design with patients and caregivers must become standard practice, not an afterthought. Technologies that are not perceived as usable, trustworthy, or culturally acceptable will fail to achieve impact regardless of technical merit.

### Translational readiness and commercial scale-Up

From a translational standpoint, the gap between prototype and product remains significant. Scalability, regulatory approval, and economic viability are key hurdles that must be addressed upfront.

Standardizing nanoparticle platforms—with modular designs, reproducible manufacturing protocols, and prevalidated safety profiles—can help streamline scale-up and facilitate regulatory review. Similarly, AI models used in clinical trials must meet explainability standards and integrate real-world data to support adaptive trial designs and ongoing post-market monitoring.

Flexible clinical trial frameworks that incorporate wearable sensors and decentralized data capture are also needed to validate nano-AI platforms in diverse patient populations and care settings.

Commercial success will depend on demonstrating not only clinical benefit but also cost-effectiveness and system integration. This will require investment from both public and private sectors, along with policy support for equitable access. Pricing models, reimbursement schemes, and IP frameworks must be carefully aligned with societal goals to prevent deepening healthcare disparities.

### Toward a transformative future in neurology

The vision of AI-enabled, nanomedicine-powered neurodegenerative care is no longer speculative. With proof-of-concept studies, early-phase clinical trials, and emerging integrative platforms, the foundation is already being laid for a future where real-time disease tracking, adaptive therapies, and predictive modeling move care from reactive to preventive.

However, realizing this future will take more than technical breakthroughs. It will require clinical humility—recognizing not just what is possible, but what is practical and effective. It will demand regulatory agility, capable of evaluating hybrid, learning systems that defy conventional categories. It will call for ethical vigilance, ensuring that advances prioritize safety, inclusivity, and fairness. And above all, it will require unwavering commitment to patient-centered design, where technologies serve human needs—not the other way around.

If guided wisely, the convergence of AI and nanotechnology in neurology holds the promise not only to extend life, but to meaningfully improve its quality. This transformation will depend as much on translational judgment and social responsibility as it does on scientific innovation.

## Conclusion

The convergence of nanotechnology and artificial intelligence represents a bold and necessary rethinking of how we approach NDDs—conditions that have long resisted conventional diagnostics and therapeutics. By integrating nanoscale precision with data-driven insight, these platforms offer a credible path toward shifting the clinical paradigm from reactive symptom management to proactive, personalized care.

However, this transformation will not occur by technological innovation alone. If the goal is real-world impact, nano-AI systems must be designed with clinical constraints, regulatory realities, and patient diversity in mind. Challenges such as nanoparticle biocompatibility, AI model transparency, and the lack of harmonized validation pipelines are not peripheral—they are central bottlenecks that must be explicitly addressed in both research design and deployment strategy.

Moreover, optimism must be matched with translational realism. Promising proof-of-concept studies too often falter at the thresholds of reproducibility, scalability, or health system integration. For this convergence to succeed, the field must move beyond isolated breakthroughs toward integrated, ethically grounded, and economically viable platforms.

This will require more than interdisciplinary collaboration—it will require shared accountability across scientists, clinicians, engineers, regulators, patients, and policymakers. Together, we must define success not just by technical feasibility but by clinical utility, societal benefit, and equitable access.

If pursued with rigor and foresight, nano-AI technologies could indeed reshape the landscape of neurodegenerative care—offering earlier diagnoses, smarter interventions, and renewed dignity for patients navigating some of medicine’s most intractable conditions.

## Data Availability

No datasets were generated or analysed during the current study.

## Abbreviations

Aβ: Amyloid-beta
AD: Alzheimer’s disease
AI: Artificial intelligence
ALS: Amyotrophic lateral sclerosis
BBB: Blood–brain barrier
CNNs: Convolutional neural networks
CRISPR/Cas9: Clustered Regularly Interspaced Short Palindromic Repeats/CRISPR-associated protein 9
CSF: Cerebrospinal fluid
CT: Computed tomography
DL: Deep learning
DTI: Diffusion tensor imaging
GANs: Generative adversarial networks
GNNs: Graph neural networks
MCI: Mild cognitive impairment
ML: Machine learning
MRI: Magnetic Resonance Imaging
MS: Multiple sclerosis
NDDs: Neurodegenerative diseases
NfL: Neurofilament light chain
NPs: Nanoparticles
PD: Parkinson’s disease
PET: Positron emission tomography
ROS: Reactive oxygen species
SERS: Surface-enhanced raman scattering
